# Experimental data from ice basin tests with vertically sided cylindrical structures

**DOI:** 10.1016/j.dib.2022.107877

**Published:** 2022-01-28

**Authors:** Hayo Hendrikse, Tim C. Hammer, Marnix van den Berg, Tom Willems, Cody C. Owen, Kees van Beek, Nick J.J. Ebben, Otto Puolakka, Arttu Polojärvi

**Affiliations:** a Faculty of Civil Engineering and Geosciences, Department of Hydraulic Engineering, Delft University of Technology, Delft 2628 CN, the Netherlands; b Siemens Gamesa Renewable Energy, The Hague, the Netherlands; c School of Engineering, Department of Mechanical Engineering, Aalto University, P.O. Box 14300, Aalto FI 00076, Finland

**Keywords:** Ice-induced vibration, Offshore wind turbine, Forced vibration test, Crushing, Intermittent crushing, Frequency lock-in, Real-time hybrid testing, Ice-structure interaction

## Abstract

Basin tests were performed at the Aalto Ice Tank to gather data on ice-structure action and interaction from ice failing against a vertically sided cylindrical pile. The tests were performed with a real-time hybrid test setup, which combined physical and numerical components to simulate a range of test structures in real-time. The dataset includes results from tests with offshore wind turbine structures, structural models representing a series of single- and multi-degree-of-freedom oscillators, and scaled dynamic models of the Norströmsgrund lighthouse and the Molikpaq caisson structure. In addition, forced vibration tests and rigid structure tests were performed. Ice loads and structural response were measured with accelerometers, displacement sensors, potentiometers, strain gauges and load cells and the ice-structure interaction process was filmed from three different camera angles. The resulting raw data have been categorized and stored as unfiltered time series. A total of 259 different tests are included in the dataset. The model ice formation procedure and the test temperature were aimed at creating model ice that mimics the material behavior of full-scale saline ice during crushing failure, with a specific focus on the transition from brittle to ductile behavior. The data can be used for validation of models for dynamic ice-structure interaction. The offshore wind turbine data can be used to study the effect of wind loading on the interaction with ice and the effect of the specific dynamic properties of wind turbine structures with monopile foundations on the ice-structure interaction process. The forced-oscillation data can be used to quantify the time and speed dependant aspects of ice loading. The Norströmsgrund lighthouse and the Molikpaq data can be used as a reference comparison to full-scale data on ice loads.

## Specifications Table

**Table:** 

Subject	Ocean and Maritime Engineering	
Specific subject area	Ice loads and ice-structure interaction processes of vertically sided structures interacting with drifting ice that fails in crushing.	
Type of data	Table Raw time series Video recordings	
How the data were acquired	Strain gauges	FLAB-6-11, ALTHEN BV Sensors & Controls, 2288 EL Rijswijk, The Netherlands. Placed in two rings, with 8 stain gauges per ring.
	Potentiometers	LCP8S-10-10K, ETI Systems, Carlsbad CA 92008, USA
	Magneto strictive displacement sensors	BIW0007-BIW1-A310-M0250-P1-S115, Balluff B.V., 5232 BC ‘s-Hertogenbosch, The Netherlands
	Load cells	VST5000 S-type load cell, HENK MAAS Weegschalen B.V., 4264 AW Veen, The Netherlands
	Accelerometers	ADXL326, analog Devices Inc., Wilmington MS 01887, USA
	Cameras	GoPro Hero 9
	MP3	In-house control, visualization and data acquisition program, Delft University of Technology.
Data format	Raw	
Description of data collection	Basin tests were performed at the Aalto Ice Tank to gather data on ice-structure action and interaction. A real-time hybrid test setup was mounted to a carriage on a bridge spanning the ice tank. A vertically sided cylindrical pile was moved through the ice by moving the carriage along the bridge. The dynamic response to the measured ice loads was simulated by the real-time hybrid test setup for a range of test structures including offshore wind turbines, a series of single- and multi-degree-of-freedom oscillators, the Norströmsgrund lighthouse and the Molikpaq caisson structure. In addition, ice loads were measured in forced vibration tests and while moving the rigid pile through the ice with a constant speed. The model-scale ice was created by spraying a fine water mist layer by layer on the water surface. The spraying water was doped with an ethanol content of 0.3%.	
	The dataset described in the article is a subset of all collected data. The dataset consists of the experimental data for which there are no major known issues compromising the validity of the data.	
Data source location	• Institution: Aalto University • City/Town/Region: Espoo • Country: Finland	
Data accessibility	Repository name: 4TU.ResearchData Data identification number: https://doi.org/10.4121/17087462.v1 Direct URL to data: https://data.4tu.nl/articles/dataset/Data_from_ice_tank_tests_with_vertically_sided_structures_collected_during_the_SHIVER_project/17087462	

## Value of the Data

• This dataset can be used to develop and validate models for ice action and ice-structure interaction, which can be applied in the design of offshore structures subjected to ice loading.
• Researchers studying ice action and ice-structure interaction and practitioners designing offshore wind turbines for ice loading can benefit from this dataset.
• The dataset can be used for numerical model validation, for understanding the effect of wind loading on the interaction of an offshore wind turbine with drifting ice, for defining trends in the interaction with ice as a function of structural properties and for studying model-ice behavior in compressive cyclic or constant speed loading.

## 1 Data Description

**Fig. 1:**
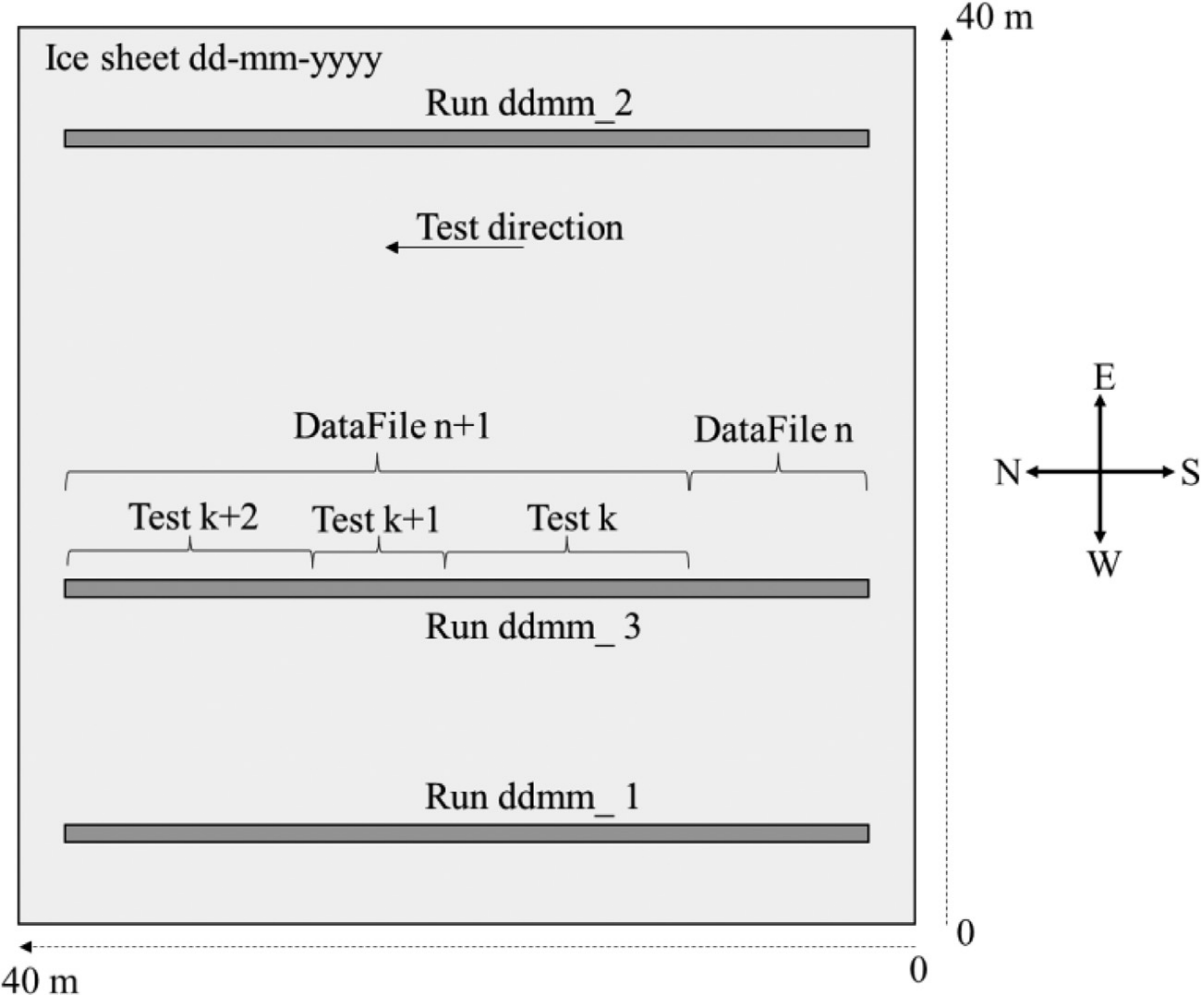
Top-view sketch of an ice sheet, showing dimensions, position conventions, test direction and numbering system for sheets, runs, data files and tests.

The dataset contains structural response and ice load data from indentation tests in ice with a cylindrical structure performed at the Aalto Ice Tank. Data from eight ice sheets are included in the dataset. Fig. 1 gives an overview of the test data and numbering conventions in relation to an ice sheet. It also shows the positioning conventions and the test direction used in all tests.

In each ice sheet, between one and 12 runs were made. A run is a single passing of the carriage from the South side of the ice basin to the North side of the basin. During a single run, one or several data recordings were made. The continuous data recordings are stored in 44 *data files* contained in the CSVDataFiles.zip archive. The ice sheets are identified by their creation date. The runs are numbered based on the run order in each individual ice sheet. The data files are numbered consecutively based on the order of recording. The tests are classified by a unique numeric identifier, numbered consecutively based on the ingestion date of the test data into a database.

Each data file may contain several tests with different test conditions. The data recorded for each test is stored separately in *test files* contained in the CSVTestFiles.zip archive*.* Test files are subsections of data files and contain continuous time series measurements under constant or linearly varying (in the case of carriage speed) test conditions. A total of 259 unique test files are included in the dataset which are divided over six different test types as shown in Table 1. A description of the test types is included in Section 3.1.

**Table 1:** Test types and corresponding unique test IDs in the CSVTestFiles.zip archive.

Test type	Corresponding unique test ID ranges
Tests with a scaled model of an offshore wind turbine in idling without wind	579–603, 662, 663
Tests with a scaled model of an offshore wind turbine in power production at rated wind speed	381–398, 400–408, 577, 578, 620–631, 634–638, 659, 661
Multi-degree-of-freedom (MDOF) structure tests	604–619
Tests with a scaled model of the Norströmsgrund lighthouse [1]	450, 453, 454
Tests with a scaled model of the Molikpaq cassion [2]	448, 449
Single degree-of-freedom (SDOF) structure tests	409–416, 441–447, 455–459, 461–465, 477, 478
Forced vibration tests	428–440, 497–552
Rigid structure tests	85, 87, 88, 90, 92, 94, 96, 118–120, 197, 200, 204, 206, 208, 210, 212, 225, 258, 260, 272, 280, 315, 336, 338, 340, 342, 364, 379, 380, 418–427, 466–476, 488–496, 571, 632, 633, 656, 657

Each data- and test file contains 36 columns of unfiltered (raw) time series data. The readme.pdf file defines the file format. The 36 columns consist of: one column where the date is recorded, one column with the time from start of the recording, seven columns with verification data from the numerical controller, two columns with load cell data, two columns with identified ice forces on the structure, two columns with potentiometer data, 12 columns with accelerometer output in *x, y* and *z* direction, two columns with the target displacement of the actuators, two columns with the measured displacements of the structure, one column for synchronization of data and video files, two columns with the numerical waterline displacement without forward prediction, a column with carriage speed and a column with carriage position. Except for the seven columns with verification data from the numerical controller, all other data is time synchronized and sampled with a sampling frequency of 2000 Hz.

The database.xlsx file gives a full overview of all tests included in the dataset, providing information on ice properties, carriage speed, and the type of test conducted in relation to each individual CSV test file. For the tests where structural models were implemented in the numerical part of the real-time hybrid test setup a reference is made to the structural files and wind load files used for this purpose. The structural files define the dynamic properties of the structures simulated by the real-time hybrid test setup and are included in the StructuralFiles.zip archive. The wind load files contain the time-domain wind loads applied at the rotor-nacelle-assembly of (some of) the offshore wind turbine structures during the tests and are included in the WindFiles.zip archive. The file format for both these file types is provided in the readme.pdf.

Video recordings in 1080p were made for each run from three different view-points: *x*-view, *y*-view, and table-view. The video recordings are divided into one file per viewpoint per run and divided over 20 zip-archives to avoid overly large file sizes. The camera locations are shown in Fig. 12.

**Fig. 2:**
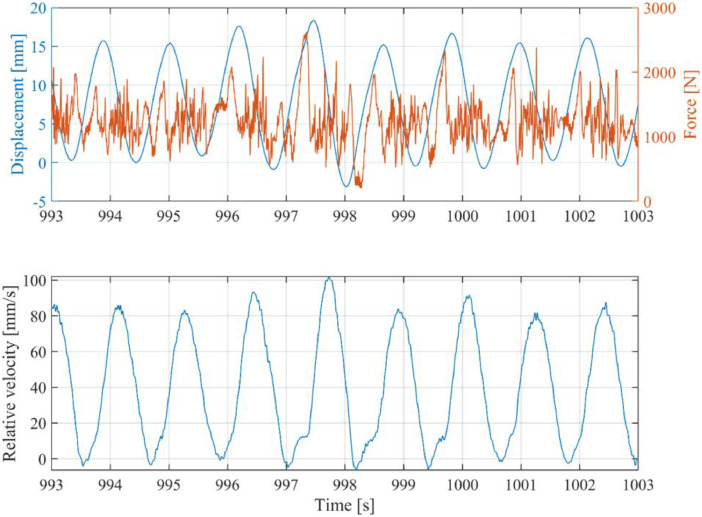
Example plot of time series data of test with ID 410, showing the time series of structure waterline displacement, the measured ice force, and the relative velocity between ice and structure. The code used to create this plot is included in the example_plot.zip archive.

Fig. 2 shows an example of how the test data can be visualized. It shows part of the test with ID 410, in which a SDOF structure was tested. The plot shows time series of the measured pile displacement in y-direction (DisplY_mm), corrected for the initial offset which can be found in the database.xlsx file, against the ice force measured by the strain gauges (FPileY1_N), and the relative velocity between ice and structure. In Fig. 2, the direction of ice drift is taken as positive. This is opposite to the coordinate system used during the tests. Thus, the recorded ice force in the dataset is generally negative. The code used to create this plot is included in the dataset in the example_plot.zip archive.

## 2 Experimental Design, Materials and Methods

The experiments were designed with the main aim to develop a model-scale benchmark dataset for offshore wind turbines loaded by crushing ice. In addition to this, tests with single-degree-of-freedom (SDOF) oscillators, existing structures for which ice-structure interaction data is described in literature, forced vibration tests and rigid structure tests were included to improve the fundamental understanding of the ice-structure interaction processes and scaling of these.

For scaling of the offshore wind turbine tests the choice was made to relax some of the scaling laws and not scale time and frequency, as the time-dependant behavior of the ice in the basin could not be easily controlled and was not known in the planning phase of the experiments. In doing so, it is assumed that the scaled loads in the basin merely occur due to the smaller geometry and strength compared to full-scale, something which requires validation. The ice speed (carriage speed in the experiment) and the waterline displacements of the test structures were kept scale-invariant. To achieve structural deformations similar to full scale, a mass scaling factor was applied to the structural models, affecting the mass, stiffness and damping. The scaling factor was determined based on the ratio between the full-scale and model-scale mean brittle crushing load:

$$\lambda_{m}=\lambda_{k}=\lambda_{C}=\frac{{\bar{F}}_{FS}}{{\bar{F}}_{MS}},$$

with $\lambda_{m}$ representing the scale factor for mass, $\lambda_{k}$ representing the scale factor for stiffness, $\lambda_{c}$ representing the scale factor for damping, ${\bar{F}}_{FS}$ the mean of the ice load during brittle crushing at high speed in full-scale and ${\bar{F}}_{MS}$ the mean of the ice load during brittle crushing at high speed in the ice tank.

This assures that the deformation of the structure in model-scale is equivalent to the full-scale deformations, consistent with the assumption that the length parameter controlling the ice failure does not change (much) between full-scale and model-scale ice, provided that an appropriate method of adjusting the model ice to better represent crushing has been obtained.

### 2.1 Test types

The different test types included in the experimental campaign are briefly described in the following sections.

#### 2.1.1 Offshore wind turbine tests

The offshore wind turbine tests were performed with a scaled ($\lambda_{m}=1700$) structural model representing a state-of-the-art offshore wind turbine on a monopile foundation designed for representative conditions in the Southern Baltic Sea. Global bending modes up to 20 Hz were included in the structural model. The tests were conducted using a lumped mass model, i.e., not explicitly modeling the turbine and blades, either responding only in the ice drift (y) direction to the ice load or in both *x*- and *y*-direction. Conditions without external wind loading, representing an idling turbine, and conditions with external wind loading aligned with the ice and under 30° and 90° misalignment were tested. For the latter conditions a generic wind load file was created containing a time series of the thrust force on the turbine at the top of the tower. This wind load was then applied to the numerical model in the modal domain.

#### 2.1.2 Multi-degree-of-freedom (MDOF) tests

MDOF tests were performed to obtain data on the dependency between the dominating vibration mode in the response of the structure and the mode shape amplitudes at water level during dynamic ice-structure interaction. Four different scenarios were tested during the test campaign. The base case consists of the first four global bending modes of an offshore wind turbine. In the second scenario, the structural mode shape amplitudes were adjusted such that the mode shape amplitude of the second global bending mode exceeds the third global bending mode by a factor 10 ($\phi_{2;y}=\;10\phi_{3;y}$). The absolute values of the mode shape amplitudes were adjusted to keep the total stiffness of the structure constant. In the third scenario, the adjustment of mode shape amplitudes was applied reversely ($10\phi_{2;y}=\;\phi_{3;y}$), while for the last scenario, mode shape amplitudes were adjusted to be equal ($\phi_{2;y}=\;\phi_{3;y}$).

#### 2.1.3 Tests with scaled structural properties of existing full-scale structures

The test setup employed in the campaign allowed to test scaled versions of existing structures for which full-scale data have been obtained in the past. The existing structures which were tested are the Norströmsgrund lighthouse and Molikpaq caisson structure [1], [2], which are well-known within the ice engineering community. As a basis for the structural models, we used the definitions in Hendrikse and Nord [3] for the Norströmsgrund and the values given by Kärnä et al. [4] for the Molikpaq. Tests adopting a carriage speed ramp were performed. Scale factors applied were $\lambda_{m}=75000$ for test 448, $\lambda_{m}=25000$ for test 449 and $\lambda_{m}=2000$ for test 450, 453 and 454.

#### 2.1.4 Single degree-of-freedom (SDOF) tests

SDOF tests were performed for the first three global bending modes of a monopile offshore wind turbine separately. The separate offshore wind turbine modes were tested to assess, by comparing the SDOF results to the multi-modal offshore wind turbine test results, to what extend the interaction of multiple modes influenced the ice-structure interaction process. In addition, using the second global bending mode of an offshore wind turbine as a base case, variations in stiffness, damping and mass were tested to investigate the influence of these parameters on the ice-structure interaction process. In addition, simulations were performed with varying mass and stiffness but with the same natural frequency. The simulated parameter combinations were chosen such that sets of three tests could be constructed in which only one parameter was varied. All SDOF structures were tested with a linearly varying carriage speed profile (*ramp*). After the ramp test, some structures were also tested at constant carriage speeds to further investigate interaction phenomena observed during the tests with a linearly varying carriage speed profile.

#### 2.1.5 Forced vibration tests

The forced vibration tests were executed in a similar fashion as the tests described in Hendrikse and Metrikine [5]. Part of the tests included sinusoidal imposed motion of the structure in the direction of ice drift where the parameters were chosen such that the relative velocity between ice and structure became zero during a cycle of oscillation. The tests performed included a repetition of those reported in the earlier work for comparison between basins and ice types. The test matrix was further expanded to smaller amplitude oscillations and to investigate the effect of not reaching a minimum relative velocity of exactly zero during the cycle.

In addition to this type of experiments which has been performed before, a first attempt to gather data allowing to study the effect of motions perpendicular to the ice drift direction on the global ice load was made. A range of amplitudes and frequencies was tested for this purpose both without- and in combination with imposed motion in the direction of ice loading at different phase angles.

#### 2.1.6 Rigid structure tests

Rigid structure tests were performed to assess the effect of ice drift speed on the ice loads. Rigid structure tests were performed at carriage speeds ranging from 0.1 to 150 mm/s. For most speeds, the rigid structure tests were repeated on several days, in several different ice sheets.

### 2.2 The Aalto ice tank

**Fig. 3:**
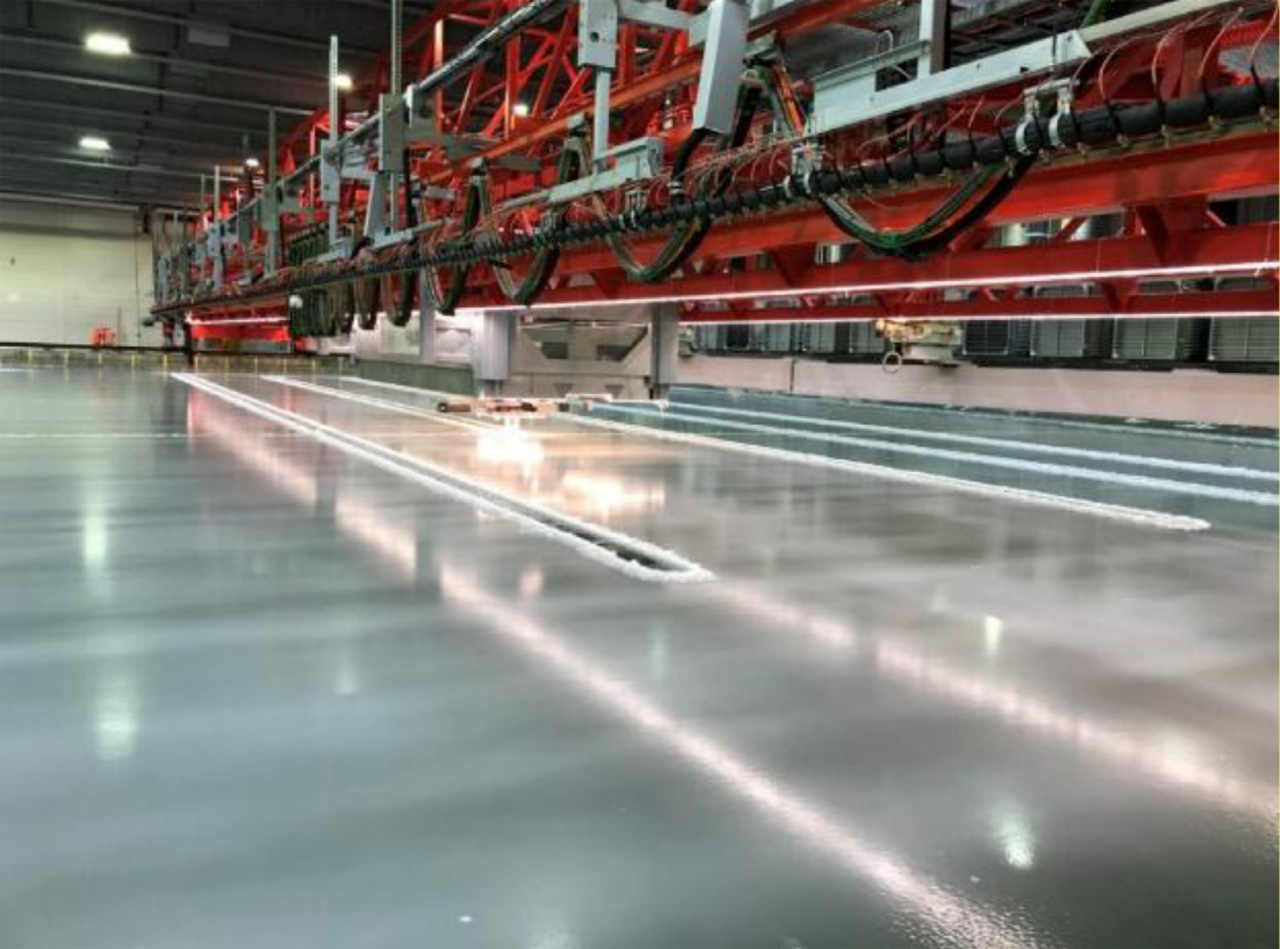
Overview image taken during the experiments, showing the bridge spanning the ice tank and the carriage mounted under the bridge.

All tests were performed at the Aalto Ice Tank. The Aalto Ice Tank is an indoor testing facility that is part of the Department of Mechanical Engineering at Aalto University. The tank consists of a 40 by 40 m basin with a depth of 2.8 m. The basin is spanned by a bridge, under which a carriage is mounted above the basin surface. The tank can operate with freshwater ice or with ethanol-doped model ice. Ethanol ice was used in the tests described in this article. Fig. 3 shows an overview image taken during the experiments, showing the bridge and carriage and the real-time hybrid test setup mounted to the carriage.

### 2.3 Model ice

On June 1st a standard model ice sheet was grown with a target thickness of 50 mm and a bending strength of 150 kPa. On June 4th the ice growing procedure was adjusted, increasing the number of freezing degree hours to 150 to obtain harder ice. However, this ice quickly lost its hard and brittle behavior as the temperature in the basin was raised to –2.5 °C. Therefore, the basin hall temperature during testing was adjusted to –11 °C on June 8th. This exceptional choice was made to maintain the desired ice failure behavior through the test day. The air temperature was further lowered to –15 °C after 15:00 on June 8th as some reduction in ice strength was observed. On the 11th, 15th, 17th, the 21st and 23rd of June the model ice was formed using a hardening time of 150 freezing degree hours and keeping the basin hall air temperature at –11 °C during the experiments. As these tests were conducted with thinner ice than those on the 8th of June there was no need to further lower the temperature during the day.

**Fig. 4:**
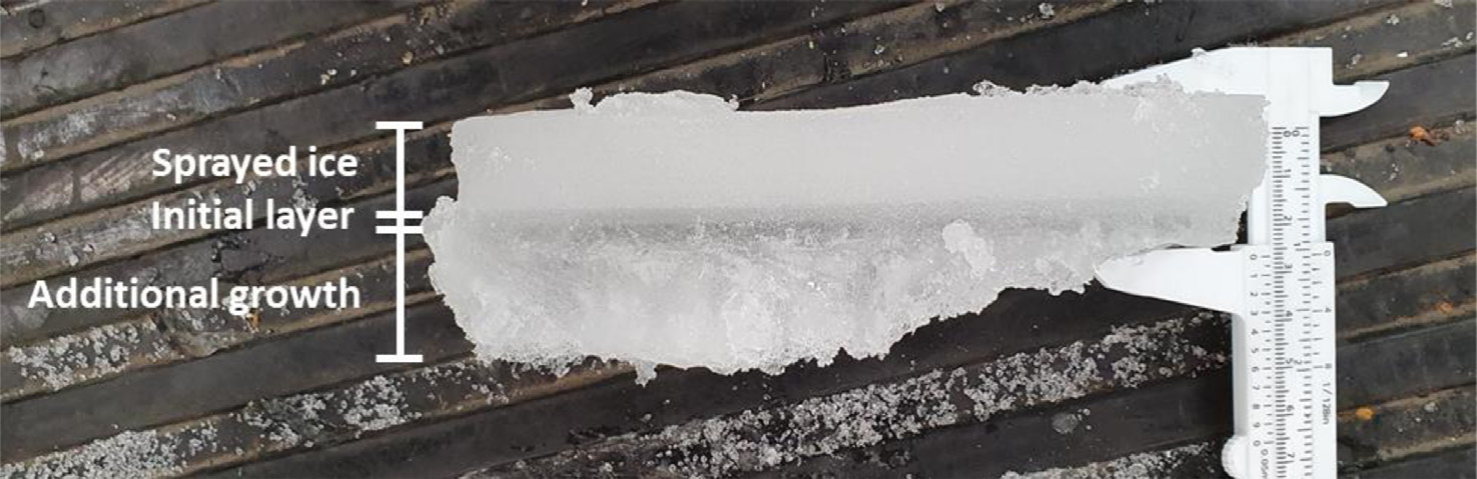
Ice vertical cross section at the end of a test day (17-6-2021): a sprayed layer on top, a layer of initial ice that was formed before the start of the spraying process in the middle, and a layer of additional ice growth at the bottom, which increases during the day.

The exceptionally low hold temperature of –11 °C resulted in substantial additional ice growth under the proper model ice layer during the experiments. This undergrowth can be observed in Fig. 4 showing a vertical cross section of the ice tank ice at the end of a test day. The top layer of the ice is formed by the spraying process. In the middle of the ice cross section a layer is visible which was the initial layer of ice on which the sprayed ice was formed. The bottom layer consists of additional ice growth. The additional ice growth mostly consisted of slushy dendritic crystal growth with little apparent ice strength. However, the top of the additional growth layer would strengthen during the day, leading to a mild increase in measured ice loads on the structure and an increase in the measured ice thickness.

**Fig. 5:**
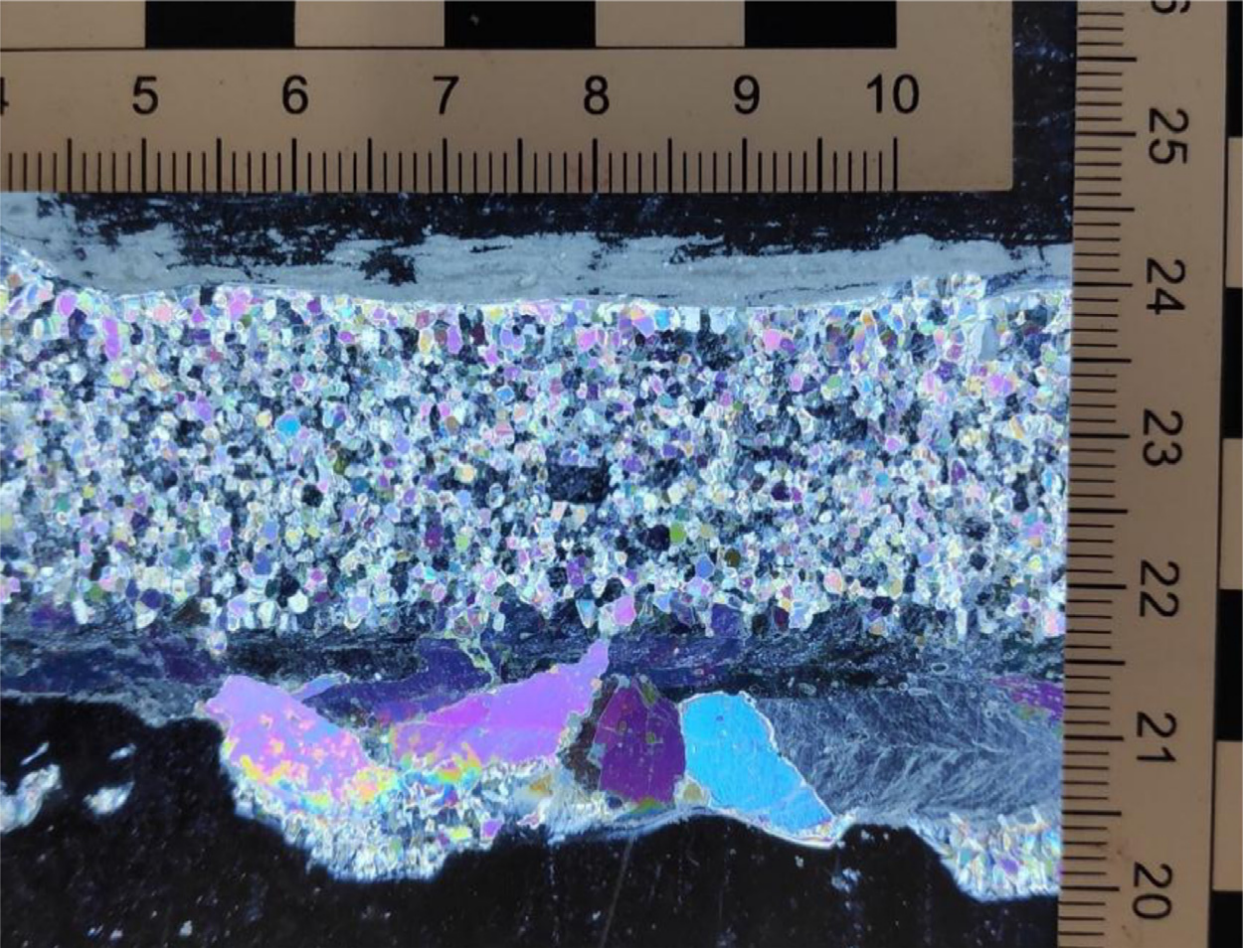
Thin section of a vertical ice cross section. The grain size in the top layer (formed by the spraying process) is on the order of ∼1 mm. The grain size in the bottom layer (natural growth) is on the order of ∼10 mm.

Fig. 5 shows the grain structure of the top (sprayed) layer and the bottom layer. The grain size in the sprayed layer was ∼1 mm. The grain size in the undergrowth was ∼10 mm.

**Fig. 6:**
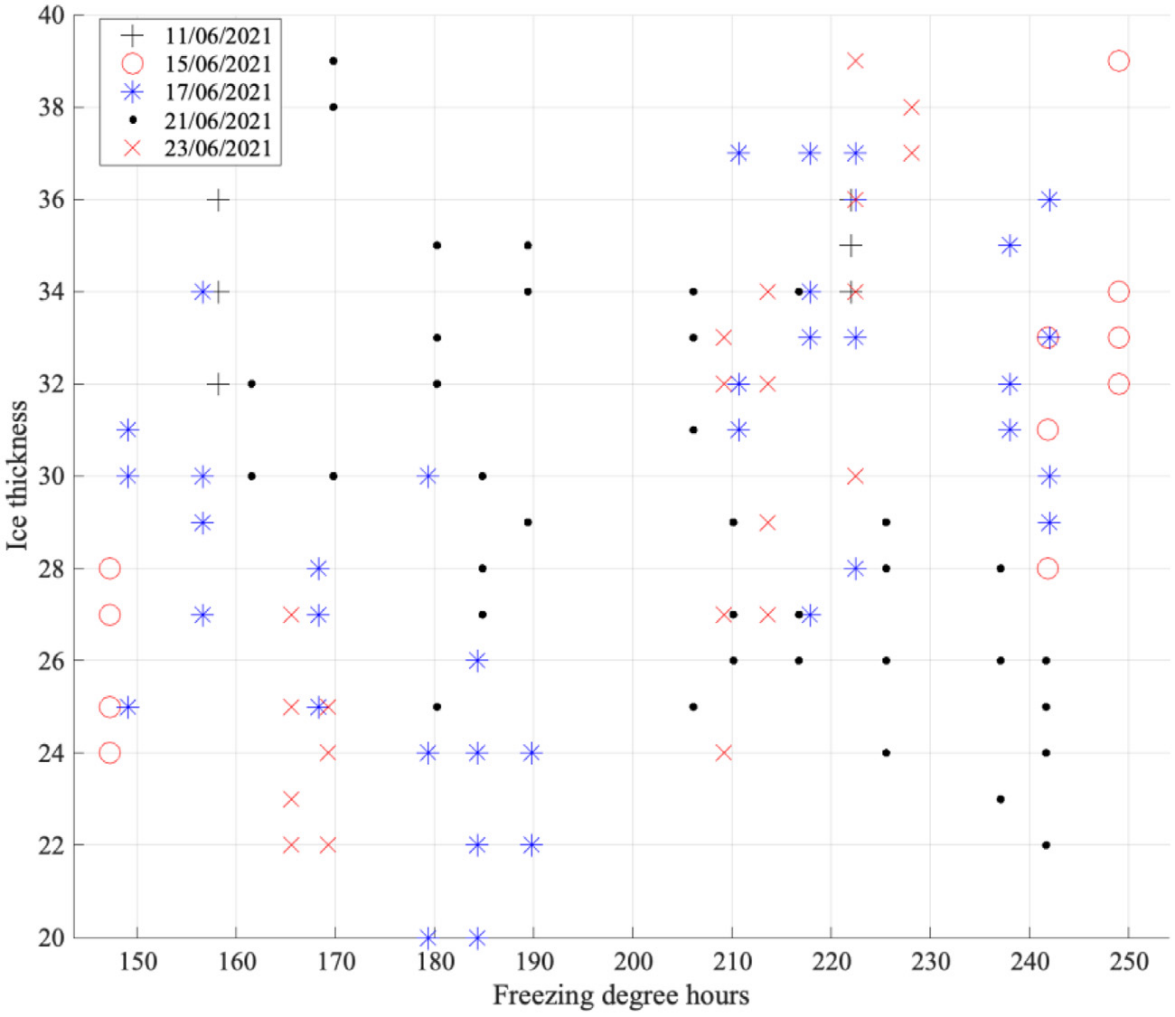
Ice thickness as a function of freezing degree hours for different test days. The ice was sampled at four locations along a tested channel at the end of a run. The ice thickness showed variability across the basin and during the day.

Fig. 6 provides an overview of the variability in ice thickness over the freezing degree hours for different test days. Thickness measurements were conducted after each run. A considerable variability in thickness was observed for different locations in the basin and during the test day. As the ice strength was only tested once a day at the end of the day trends during the day cannot be recovered from the data. For the tests between the 11th of June and the 23rd of June the tests were conducted around 18:00 (after the last run) resulting in an average compressive strength of 580 kPa (501 kPa ∼ 658 kPa) and an average bending strength of 461 kPa (374 kPa ∼ 521 kPa). For each individual test the database file can be used to obtain the relevant ice properties.

**Fig. 7:**
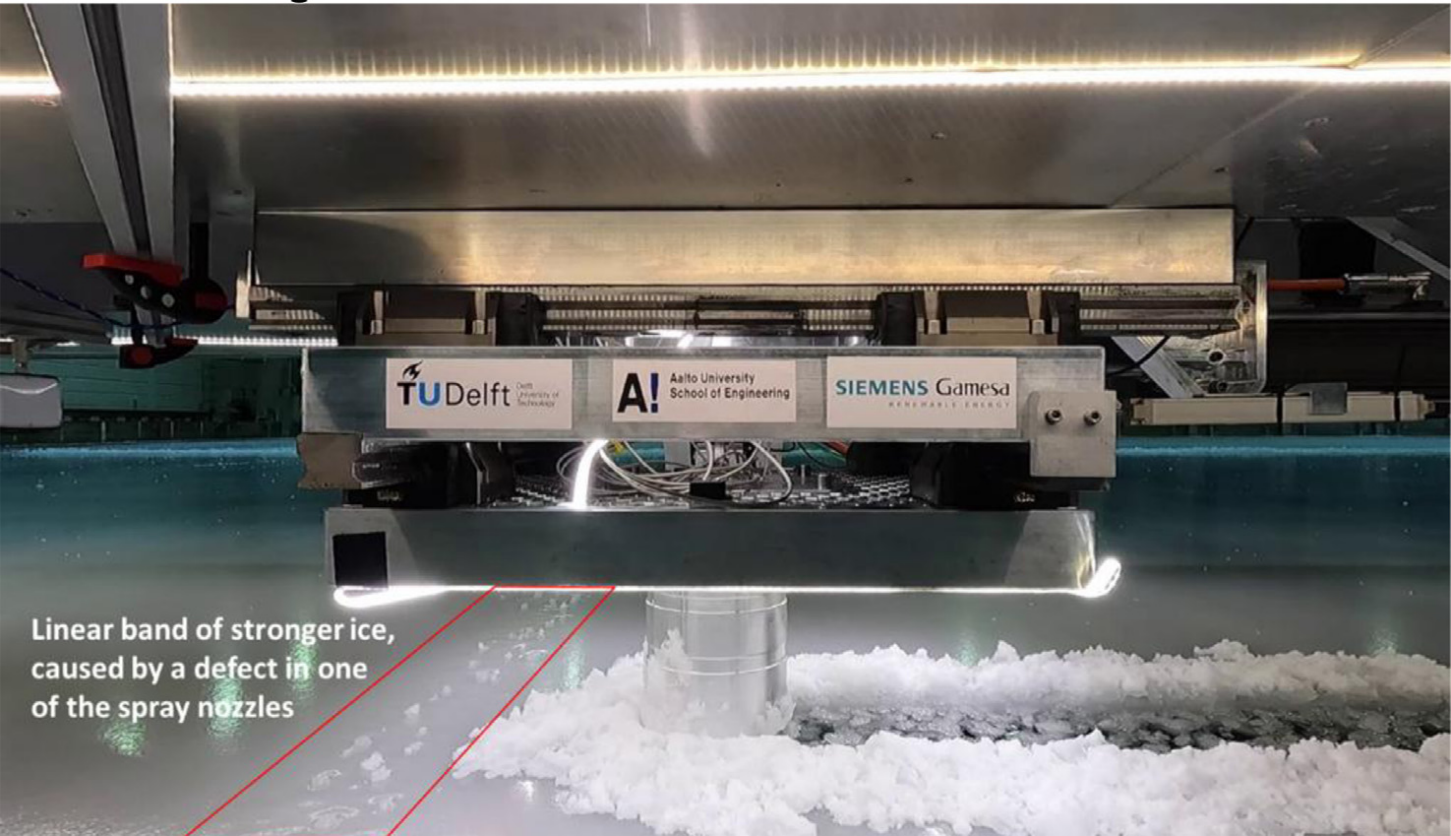
Linear band of stronger ice, formed by a defect in one of the spray nozzles.

In all tests on and before 17-6-2021, a linear band of stronger ice would run across the tank in East-West direction. This band was caused by a defect in one of the spraying nozzles, and was fixed in the tests after June 17th. The band of stronger ice occurred at a carriage position of 21.7 m (as recorded in the CarPos_m column in the test data files). An increase in ice load can be observed in the test data at this carriage position for the tests performed on and before June 17th. Fig. 7 shows the band of stronger ice as observed in the video recordings of the tests.

### 2.4 Real-time hybrid test setup

**Fig. 8:**
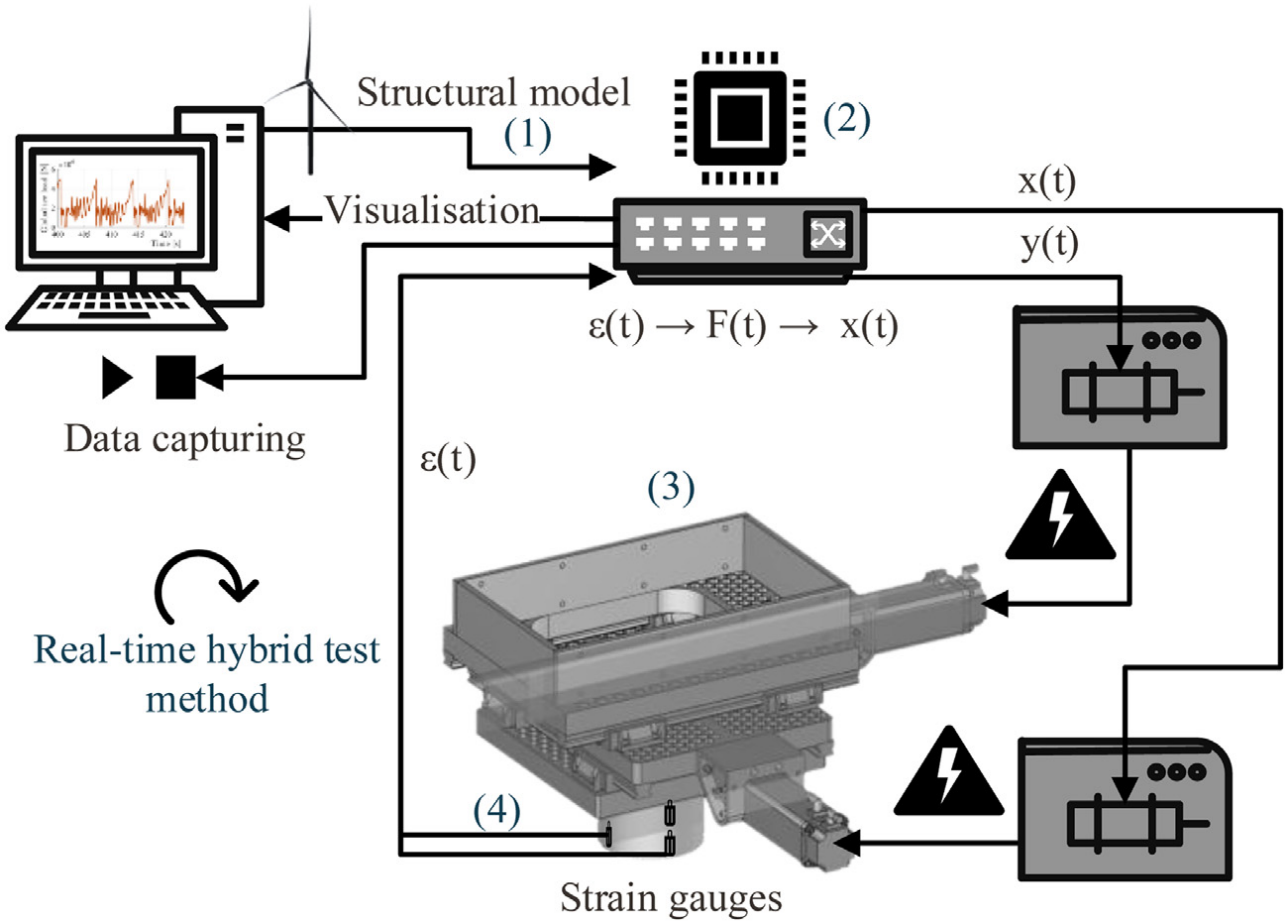
Overview of the real-time hybrid test setup (from [6]).

The tests were performed with a real-time hybrid test setup which combines physical and numerical components to simulate the test structure in real-time. Fig. 8 shows an overview of the test setup and Fig. 7 shows the setup connected to the carriage during operation. The structural model was simulated numerically on a microprocessor. The waterline displacement calculated by the numerical model was sent to bi-directional linear actuators which matched the numerically calculated waterline displacement on the physical test pile. The ice loads were identified in real-time on the test pile by calibrated strain gauges. The measured ice load signal was then applied to the numerical structural model, closing the real-time simulation loop. During the tests, data storage, data visualization and system control were handled by a control laptop, which was connected to the microprocessor and to the data acquisition system. Details of the setup and pre-testing are provided in Hammer et al. [6]. Here, only the aspects relevant for understanding and interpretation of the data are included.

#### 2.4.1 Pile, tables, actuators and sensors

**Fig. 9:**
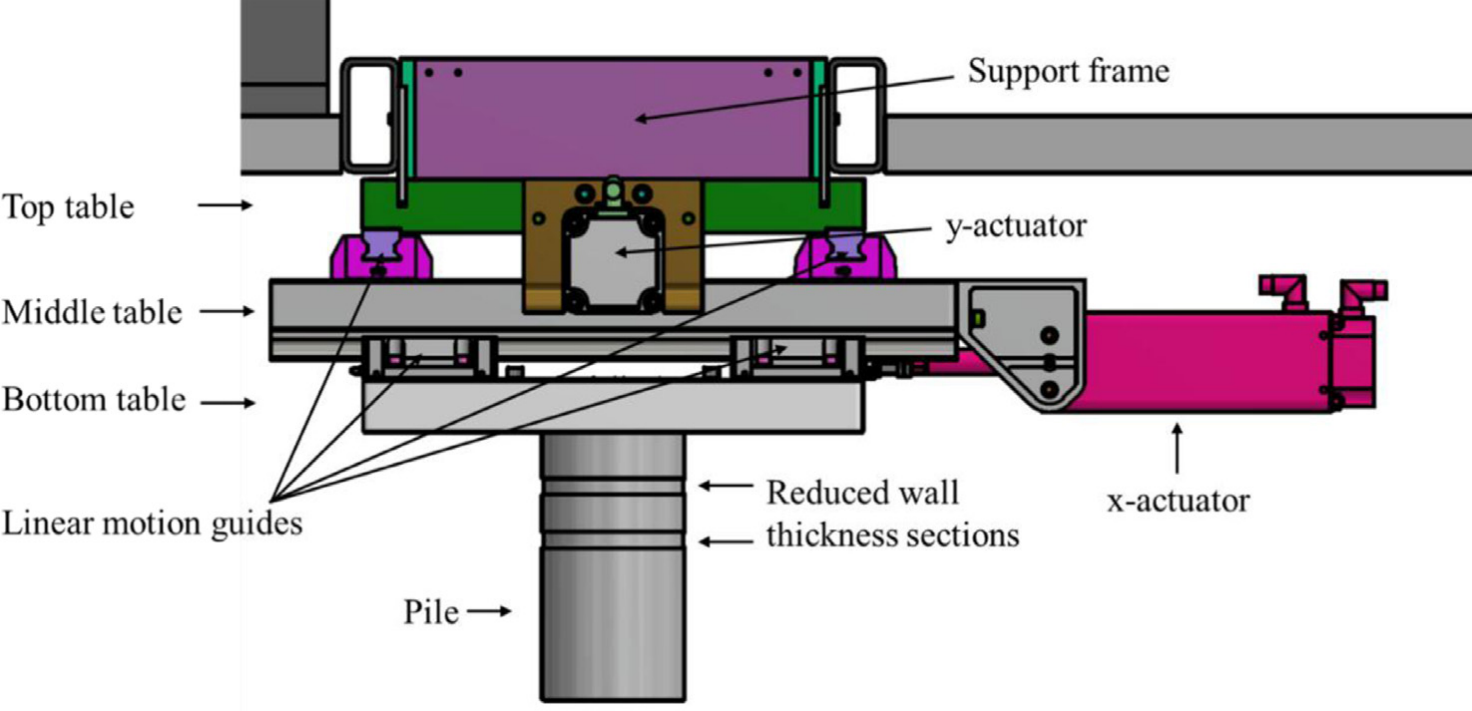
Side-view sketch of the real-time hybrid test setup.

Fig. 9 shows a side-view sketch of the real-time hybrid test setup. The mechanical parts of the test setup consisted of a pile interacting with the ice, a bottom table moving in *x*-direction relative to the middle table, a middle table moving in *y*-direction relative to the carriage, and a top table which is fixed to the carriage through a support frame. The pile was attached to the bottom table by a bolted connection. The outer diameter of the pile interacting with the ice was 200 mm. The mass of the total setup was approximately 390 kg.

**Fig. 10:**
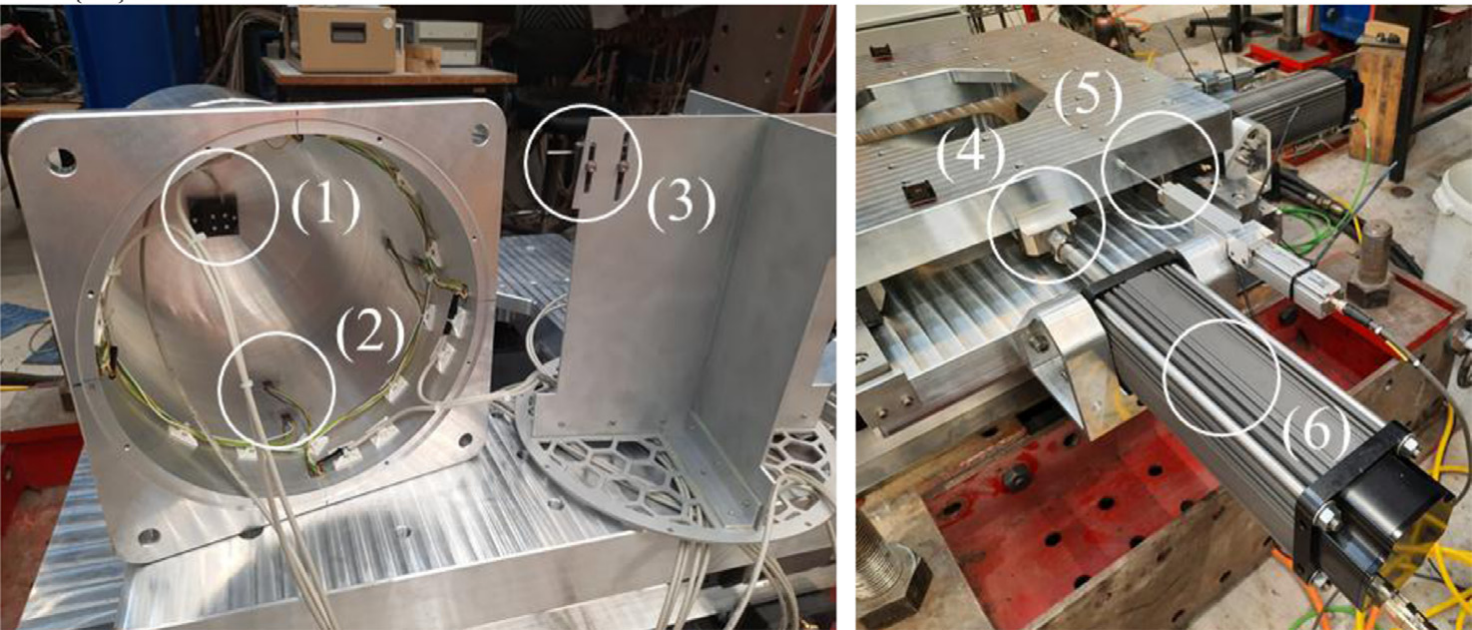
Left: Pile that penetrates the ice during tests, showing the accelerometer at the bottom of the pile (1), the strain gauges (2) and the potentiometers (3). Right: tables and actuators, showing the load cells (4), the displacement sensors (5) and the actuators (6). The load cells, displacement sensors and actuators are mounted both in x- and y-direction. (from [6]).

**Fig. 11:**
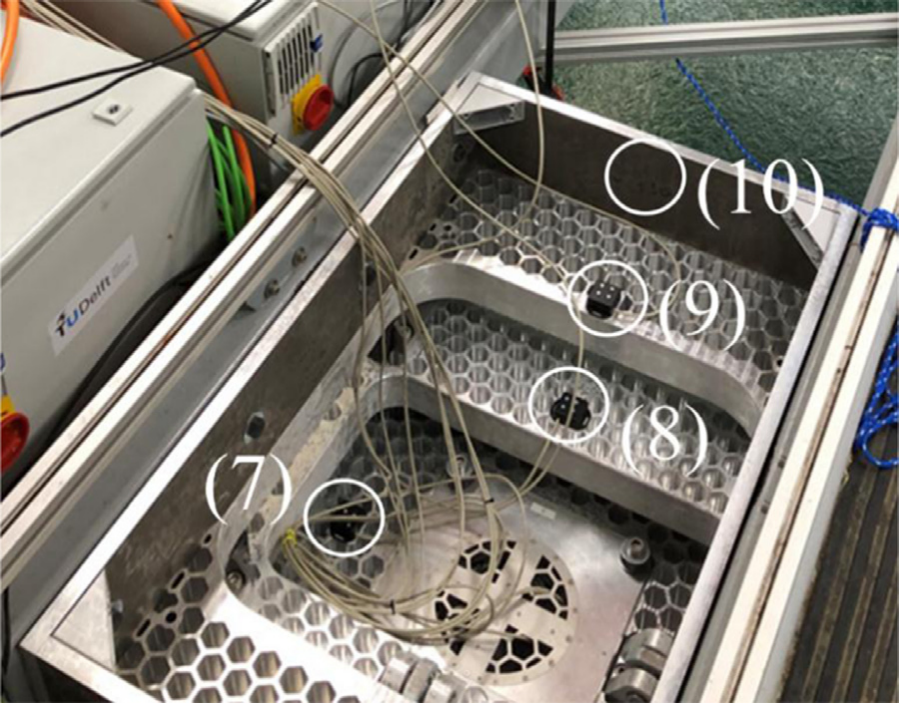
Test setup mounted to the carriage, showing the accelerometers on the bottom table (7), the middle table (8) and the top table (9). The bottom table moves in *x*-and *y*-direction as driven by the actuators. The middle table only moves in *y*-direction. The top table is fixed to the carriage through an aluminium support frame (10).

Figs. 10 and 11 show the locations of the sensors and actuators. An accelerometer was connected to the bottom of the pile (1). Strain gauges were installed above the ice action point (2), to measure the strain. The calibrated strain gauges were used to derive the ice force experienced by the pile during testing. Potentiometers (3) were mounted to a cruciform insert which was disconnected from the walls of the circular pile. The potentiometers measured the local deformation of the outer circular pile relative to the (unloaded) cruciform. At the location of the strain gauges, the wall diameter of the pile was reduced from 5 to 3 mm in order to increase the strain and improve the accuracy of the strain gauge measurements. The loads applied by the actuators to the tables were measured by load cells (4). The displacements of the bottom table relative to the middle table in *x*-direction, and of the middle table relative to the top table in *y*-direction were measured by displacement sensors (5). The table movement in *x*- and *y*-direction was driven by linear actuators (6). Accelerometers were mounted to the bottom (7) middle (8) and top (9) tables. The entire setup was connected to the carriage by an aluminium support frame (10).

#### 2.4.2 Cameras

**Fig. 12:**
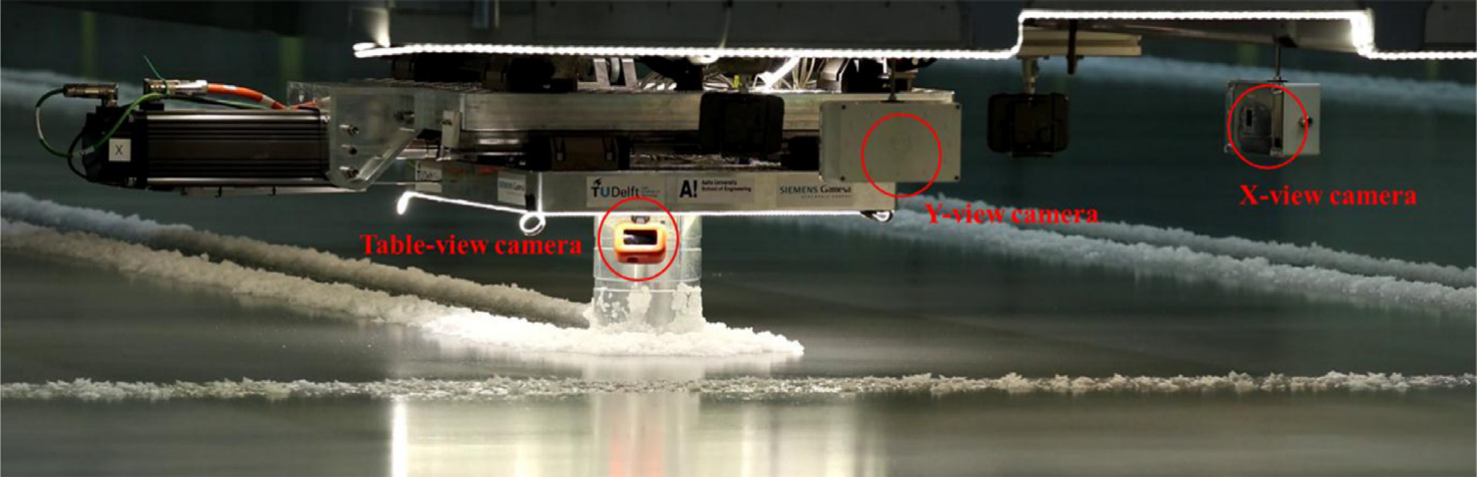
Cameras capturing the ice-structure interaction from different viewpoints.

All tests were captured by GoPro Hero 9 cameras from three different viewpoints: Table-view, *X*-view and *Y*-view. The camera positions are shown in Fig. 12.

#### 2.4.3 Control system

The numerical component of the real-time hybrid test setup was handled by a Teensy USB Development Board (Version 3.6, PJRC). In real-time, this microcontroller received the voltage signal from the upper ring of strain gauges, converted the signal to a load based on a calibrated conversion factor, calculated the structure response to the measured load, and sent the target position to the actuators based on the numerically calculated waterline position of the structure.

##### 2.4.3.1 Structure representation

Structures were represented in the numerical model by single- or multi-degree-of-freedom linear models in the modal domain. Note that the equations of motion presented in [1] miss the coupling terms between x and y direction which were introduced in the software prior to testing. The dynamic properties of a structure were defined by the frequency, damping (logarithmic decrement) and mass-normalized modal amplitudes in x- and y-directions at the ice action point of each structure mode that was considered. In addition, the modal amplitude at points other than the ice action point could be specified to enable the consideration of external load effects, such as the effect of wind loads on offshore wind turbines. Damping values could be ramped from a start to an end value. This functionality was used in some tests to mitigate the effect of initial conditions on the dynamic interaction.

**Fig. 13:**
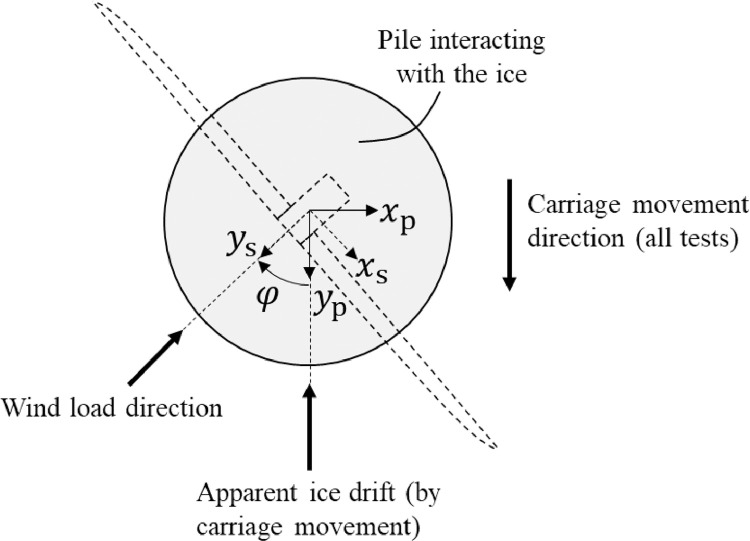
Physical test pile ($x_{p},\;y_{p}$) and numerical structure ($x_{s},\;y_{s}$) coordinate systems, in reference to the carriage movement direction.

Fig. 13 shows the structure and test pile coordinate system in reference to the carriage movement direction. The numerical structural model coordinate system could be rotated relative to the coordinate system of the test pile. A rotation φ between the structure and test pile coordinate system was only applied in offshore wind turbine tests with wind-ice misalignment. In all other tests, the structure and pile coordinate systems were identical. The measured data are always defined in the coordinate system of the physical test pile.

##### 2.4.3.2 Time stepping scheme

The structure response was calculated in real-time using the Euler-Cromer method (also called Semi-implicit Euler method). A time step size of $10^{-4}\;s$ was used in all tests. The execution time of the time stepping function was ∼ 55µs for the structure with the highest number of modes used, and lower for structures with fewer modes, allowing for real-time execution of the numerical structure model.

##### 2.4.3.3 Actuator delay compensation

In the real-time hybrid measurement, simulation and response loop, the ice force identified from the strain gauges needs to be sent to the control system. The control system needs to calculate the structure response to the ice load. The new waterline position needs to be sent to the actuators, and the actuators must go to the updated position. Because of this process, there is an inherent delay between the measured ice load and the physical response of the structure to the measured ice load. This delay may cause inaccuracy or instability in the structural response to the ice loads. To compensate for the delay in the measurement, simulation and response loop, actuator delay compensation was applied to the positions sent to the actuators. The delay was compensated by forward predicting the structure position:

(1) $$\begin{array}{c} {\dot{w}}_{i;\text{fp}}={\dot{w}}_{i}\left(t_{j}\right)+\;\Delta t_{\text{fp}}\cdot {\ddot{w}}_{i}\left(t_{j}\right)\\ w_{i;\text{fp}}=w_{i}\left(t_{j}\right)+{\dot{w}}_{i;\text{fp}}\cdot \Delta t_{\text{fp}} \end{array}$$

where $\Delta t_{\text{fp}}$ is the forward prediction time and $w_{i;\text{fp}}$ is the forward-predicted modal displacement.

The forward prediction time was based on delay measurements performed during the initial tests of the test campaign. The delay between the numerical structure waterline position and the physical actuator position was determined by finding the maximum cross-correlation between the measured displacement and the time-shifted numerical displacement. Based on these measurements, the target position that was sent to the actuators by the control system was forward predicted by 5 ms. The directly measured pile displacement (channels DisplY_mm / DisplX_mm), the numerical structure position in the control module (channels SOutYnoFP_mm / SOutXnoFP_mm), and the forward-predicted position that was sent to the actuators (channels SOutY2_mm / SOutX2_mm) are stored separately in the test output, allowing for the evaluation of the forward prediction scheme.

### 2.5 Test procedures

This section describes procedures that were followed during the test campaign relevant to the use of the data.

#### 2.5.1 Data and video synchronization

In tests with IDs ≤ 145, the recorded data and the video files were synchronised by applying a force impulse (hammer impact) to the test setup. This force impulse is traceable in the recorded acceleration data and in the videos. In tests with IDs > 145 synchronization was facilitated by a LED blink, which is also visible in the data recording.

#### 2.5.2 Calibration of strain-gauge based load measurements

The strain-gauges were used in the experiments to identify the ice load. This required a calibration factor linking the output of the strain gauges to the ice load to be defined as the point of ice action on the pile was not known in advance of the experiments. This factor was obtained based on a comparison to the load cell data for tests with the actuators in fixed position, minimizing the inertial contribution of the test setup in the load cell data.

#### 2.5.3 Carriage speed

In most tests, the carriage to which the test setup was attached was moving at a constant, linearly varying or bilinearly varying speed. The carriage movement represents the ice drift (with the ice drift direction being opposite to the carriage movement direction). The actuators were used (with the carriage stationary) to test constant speed ice action at low speeds because this enabled a more accurate speed control. In tests at speeds of 5, 10 or 20 mm s^−1^ either the actuators or the carriage was used to move the pile. It can be derived from the test data (DisplY_mm and CarVel_mm_s data columns) which method was used to move the pile.

#### 2.5.4 Actuator offset

In most tests, an initial actuator offset from the zero-position was applied in order to prevent reaching the actuator limits during the tests. If possible, the actuator offset was set such that the actuators would not cross their zero-position during the tests, as an irregularity was observed in the force and acceleration signals when the actuators crossed their zero-position. The cause of this irregularity is currently not know and it remains in the data for those tests where the zero-position is crossed.

#### 2.5.5 Ice thickness measurements

Ice thickness measurements were performed after most test runs. The ice thickness was measured at four equidistant points along the test channel at approximately 0, 10, 20 and 30 m. The ice thickness was measured with a calliper. The slushy ice which formed at the bottom during the day was scraped off with the calliper before taking the thickness measurement.

#### 2.5.6 Material property tests

**Fig. 14:**
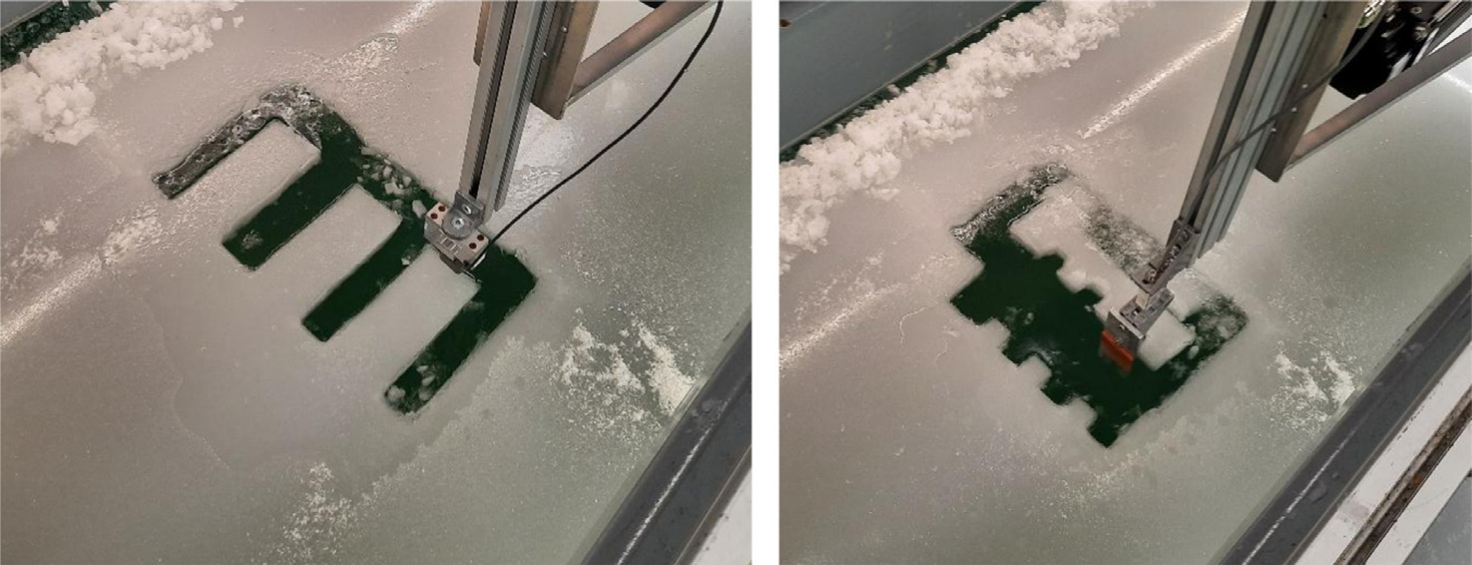
Left: Flexural strength measurements by loading ice beams with a defined length and cross-sectional area in downward bending until failure. Right: compressive strength measurements by loading protrusions with a defined area in compression until failure.

The model ice mechanical properties were determined according to the ITTC (International Towing Tank Conference) recommended procedures (7.5-02-04-02) [7]. The ice flexural strength was measured by loading cantilever beams of ice cut into a free edge of the sheet. The beams had a length of around 5 h and a width of 2 h, where h is the ice thickness; these would translate to about 133 mm and 60 mm, respectively. The beams were loaded downwards at the tip until they failed near the root (Fig. 14). The additional buoyancy force caused by the reduction in freeboard during bending was subtracted in the calculation of the flexural strength. The buoyancy force was determined as the force on the loading pin after the cantilever beam was fractured.

The compressive strength was measured by loading a series of square protrusions cut out of a free edge of the ice sheet. The protrusions had dimensions of around h×h×h, where h is the ice thickness; these corresponded to around 30 mm in most of the tested sheets. The protrusions were compressed horizontally with a flat indentor against the sheet until first failure. The indentor face was orientated flat against the protrusion face and load levelling was further promoted through the use of a 5 mm sheet of silicone rubber facing on the plate.

The Young's modulus was determined by measuring the deflection of the intact ice plate under a predefined load of 200 g. Assuming the ice sheet to behave as a linear-elastic plate on an elastic foundation, the measured deflection was used to estimate the Young's modulus.

### 2.6 Data limitations

The ice force derived from the strain gauge measurements (FPileX1_N and FPileY1_N) was used in the real-time hybrid test setup to calculate the structure response. We recommend to use this data as the primary source of ice load data in data analysis. The loads measured by the load cells (FLoadX_N and FLoadY_N) contain significant contributions from the inertia of the tables in the setup. The loads measured by the strain gauges in *x*-direction (FPileX1_N) show a drift. This drift may be corrected by comparison to the load cell data in *x*-direction.

The potentiometer signals Pm1X and Pm1X represent an ice load estimate derived from local pile displacements at two locations (see Section 3.4.1 for sensor locations). The measured data shows that the ice load derived from the potentiometer signals is inaccurate. This is thought to be caused by the fact that the sensors only measure the local relative displacement at two points along the waterline, and that the distributed ice load cannot be derived from the local displacements in an accurate manner. The potentiometer signals in the data files can be converted back to the measured displacement by multiplying the recorded signals with factors 6.9901E-6 and 4.5963E-6 for the *x*- and *y*-direction, respectively.

### 2.7 Reasons for data exclusion

The dataset described in this article covers 259 tests out of a total of 663 tests that were performed in this test campaign. The remaining tests are excluded from the dataset mainly for two reasons.

The towing carriage to which the test setup was connected was moving along rails driven by two electro-motors. The electro-motors were controlled by PID controllers with a constant or linearly varying speed target. In all data files prior to data file mode_377 (thus mode files < 377), the carriage PID controller settings were suboptimal for the highly dynamic load. The carriage was unable to maintain a constant speed during the tests, and the varying carriage speed had a significant influence on the test results. As the varying carriage speed influenced the ice-structure interaction process, this effect cannot be mitigated in post-processing. All tests in which the varying carriage speed issue occurred have been excluded from the dataset.

Twenty tests were performed with structural models of offshore wind turbines containing confidential data. Data from these tests is excluded from the publicly available dataset.

## Ethics Statements

N/A.

## CRediT authorship contribution statement

**Hayo Hendrikse:** Conceptualization, Methodology, Validation, Investigation, Data curation, Writing – original draft, Visualization, Supervision, Project administration, Funding acquisition. **Tim C. Hammer:** Conceptualization, Methodology, Software, Validation, Investigation, Data curation, Writing – review & editing. **Marnix van den Berg:** Conceptualization, Software, Validation, Investigation, Data curation, Writing – original draft, Visualization. **Tom Willems:** Conceptualization, Methodology, Software, Investigation, Data curation, Writing – review & editing, Supervision, Project administration, Funding acquisition. **Cody C. Owen:** Investigation, Data curation, Writing – review & editing, Visualization. **Kees van Beek:** Methodology, Software, Validation, Resources, Writing – review & editing. **Nick J.J. Ebben:** Validation, Investigation, Data curation. **Otto Puolakka:** Investigation, Resources, Writing – review & editing, Project administration. **Arttu Polojärvi:** Writing – review & editing, Investigation, Project administration.

## Declaration of Competing Interest

The authors declare that they have no known competing financial interests or personal relationships that could have appeared to influence the work reported in this paper.
